# Effects of ∆^9^-tetrahydrocannabinol on aversive memories and anxiety: a review from human studies

**DOI:** 10.1186/s12888-020-02813-8

**Published:** 2020-08-26

**Authors:** Ana Maria Raymundi, Thiago R. da Silva, Jeferson M. B. Sohn, Leandro J. Bertoglio, Cristina A. Stern

**Affiliations:** 1 Department of Pharmacology, Federal University of Parana, Curitiba, PR Brazil; 2 Department of Pharmacology, Federal University of Santa Catarina, Florianopolis, SC Brazil

**Keywords:** *Cannabis*, Cannabidiol, Fear extinction, THC, Memory reconsolidation

## Abstract

**Background:**

Posttraumatic stress disorder (PTSD) may stem from the formation of aberrant and enduring aversive memories. Some PTSD patients have recreationally used *Cannabis*, probably aiming at relieving their symptomatology. However, it is still largely unknown whether and how *Cannabis* or its psychotomimetic compound Δ^9^-tetrahydrocannabinol (THC) attenuates the aversive/traumatic memory outcomes. Here, we seek to review and discuss the effects of THC on aversive memory extinction and anxiety in healthy humans and PTSD patients.

**Methods:**

Medline, PubMed, Cochrane Library, and Central Register for Controlled Trials databases were searched to identify peer-reviewed published studies and randomized controlled trials in humans published in English between 1974 and July 2020, including those using only THC and THC combined with cannabidiol (CBD). The effect size of the experimental intervention under investigation was calculated.

**Results:**

At low doses, THC can enhance the extinction rate and reduce anxiety responses. Both effects involve the activation of cannabinoid type-1 receptors in discrete components of the corticolimbic circuitry, which could couterbalance the low “endocannabinoid tonus” reported in PTSD patients. The advantage of associating CBD with THC to attenuate anxiety while minimizing the potential psychotic or anxiogenic effect produced by high doses of THC has been reported. The effects of THC either alone or combined with CBD on aversive memory reconsolidation, however, are still unknown.

**Conclusions:**

Current evidence from healthy humans and PTSD patients supports the THC value to suppress anxiety and aversive memory expression without producing significant adverse effects if used in low doses or when associated with CBD. Future studies are guaranteed to address open questions related to their dose ratios, administration routes, pharmacokinetic interactions, sex-dependent differences, and prolonged efficacy.

## Background

### Posttraumatic stress disorder

The formation of intense and long-lasting aversive memories after threatening or stressful events affects the individual’s quality of life when it triggers the development of posttraumatic stress disorder (PTSD [1, 2];), which is categorized on the DSM-5 as a trauma- or stressor-related disorder [3]. After a traumatic event exposure, PTSD patients gradually present characteristic symptoms, such as increased anxiety, hyperarousal, and avoidance of cues associated with the trauma. The inappropriate expression of fear-related responses in non-risky situations is also frequent. Moreover, intrusive thoughts, nightmares, and resistance to extinguish the aversive/traumatic memory have been reported [4–6].

### Fear memory extinction and reconsolidation

Fear extinction is a form of inhibitory learning that suppresses the expression of the original aversive/traumatic memory. As a consequence, individuals express less fear responses. In both laboratory animals and healthy humans, prolonged and repeated exposures to conditioned cues without presenting the aversive stimulus can induce it [7, 8]. Preclinical studies have shown that the extinction process requires activity and plasticity in several interconnected brain regions, including the infralimbic and prelimbic subregions of the medial prefrontal cortex [homologous to the human ventromedial prefrontal (vmPFC) and dorsal anterior cingulate (dACC) cortices, respectively], and some amygdala nuclei [9–11]. Specific PTSD psychotherapies (e.g., prolonged exposure therapy) are based on extinction learning [12]. Patients suffering from this psychiatric condition, however, often present extinction impairments [10, 13] accompanied by a hypoactive vmPFC [14, 15], hyperactive dACC and amygdala [14, 16–18], and a smaller and hypofunctional hippocampus [14, 18, 19]. The abnormal functioning of these brain regions could explain not only the hyperarousal and extinction deficits but also the increased responsiveness to trauma-unrelated stimuli leading to fear overgeneralization [20–22]. Noteworthy, over time, the original aversive/traumatic memory can spontaneously reemerge, which also limits the efficacy of the extinction approach [23].

Upon recall, a consolidated memory can become labile again and, thus, its content is destabilized and gradually reconsolidated after that, being susceptible to intervention-induced changes during this period. In both laboratory animals and healthy humans, short exposure to conditioned cues can induce memory destabilization and reconsolidation [24]. The neural substrate regulating this process and that underlying extinction is thought to be overlapping, yet distinct [24, 25]. Besides, the relative contribution of a given brain region in each case may vary. For instance, the rodent prelimbic cortex (dACC in humans) is more involved in aversive memory reconsolidation than extinction [11, 25–27]. The age and intensity of the memory are factors that influence the chance of destabilization upon retrieval in both laboratory and clinical settings [28–31]. Unlike extinction, however, changes in the original aversive/traumatic memory-related outcomes are permanent; therefore, impairing fear memory reconsolidation could have value for treating PTSD [32–35].

**Fig. 1:**
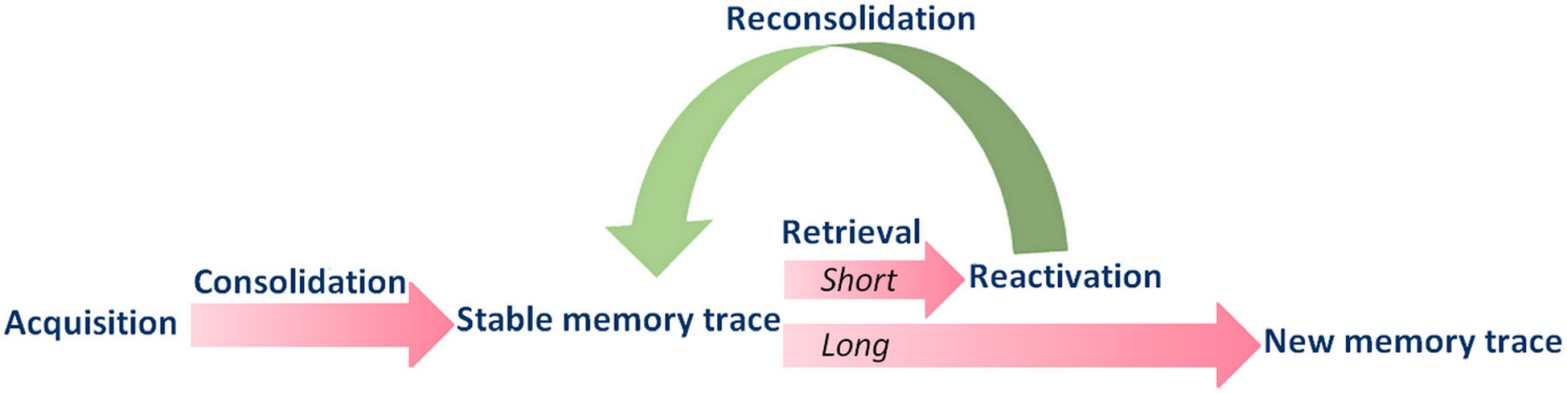
The process of aversive memory formation, extinction, and reconsolidation. Immediately after an emotionally relevant experience, the acquired memory undergoes the gradual process of consolidation. Upon retrieval, a brief but sufficient conditioned stimulus exposure event induces memory reactivation or destabilization. In other words, the stable memory trace becomes labile again and, thus, its emotional content is modifiable until the reconsolidation stage is ended. Based on this, drugs aiming at interfering with reconsolidation can be administered after memory reactivation. After a prolonged or repeated period of memory retrieval, the extinction process is triggered, leading to the formation of a new memory trace that competes and inhibits the original aversive memory, reducing fear responses. Drugs that potentiate/facilitate memory extinction are usually administered before extinction learning. Other phases (e.g., consolidation) of memory extinction can also be targeted, although it has scarcely been explored. As reviewed here, low doses of THC attenuate aversive memory expression through anxiety reduction, extinction facilitation, and reconsolidation impairment (currently shown in laboratory animals only)

In preclinical studies, fear conditioning is a standard procedure for investigating the process of fear memory extinction and reconsolidation. It has a translational value from laboratory animals to healthy humans, and onwards to anxious/PTSD patients. However, the stimuli, the primary outcome measures, and the populations typically used differ between animal and human studies [36]. Besides, whereas in humans it is possible to assess the explicit and implicit aversive memory components, in laboratory animals, only implicit memory-related behavioral, autonomic, and hormonal measures can be assessed [37]. It should also be acknowledged that a strong aversive memory is not necessarily maladaptive. It is indispensable to evaluate not only qualitative but also quantitative aspects to infer whether the selected experimental protocol has indeed simulated one or more PTSD symptoms or features [37]. Similar considerations presumably apply while testing and modeling anxiety in rodents and humans [38, 39] and, thus, the interpretation and extrapolation of basic and clinical findings are not straightforward (Fig. 1).

### Evidence for the role of the endocannabinoid system in PTSD and its treatment

A low “endocannabinoid tone” has been reported in PTSD patients. Relative to healthy controls, they present reduced circulating concentrations of anandamide and 2-arachidonoylglycerol (2-AG), and lower hair concentration of palmitoylethanolamide (PEA), oleoylethanolamide (OEA) and stearoylethanolamide (SEA) [40–42]. They also have up-regulated cannabinoid type 1 (CB1) receptor expression in the hippocampus, dACC, and amygdala, an alteration more pronounced in women than men [40]. The genetics research focusing on variants in the genes of the CB1 receptor and the fatty acid amide hydrolase (FAAH) enzyme, which metabolizes anandamide, has shown corresponding findings. A specific variant resulting in high expression of CB1 receptors can increase the risk of developing PTSD or anxiety-related disorders [43, 44]. However, studies in healthy volunteers have shown that a genetic variant resulting in a lower FAAH activity can influence stress reactivity and fear extinction, being protective against PTSD or anxiety-related disorders [45–47]. Despite the advance in understanding PTSD neurobiology, the selective serotonin reuptake inhibitors (SSRIs) are mostly used to manage PTSD symptoms, with some benefit at best. Accordingly, most reviews have concluded that the benefit and effect sizes of these drugs are small [48–50]. Besides, PTSD comorbidities include anxiety and substance use disorders, making an effective treatment with SSRIs and psychotherapies even more challenging [51].

There is evidence relating *Cannabis* use in PTSD patients to relaxation, sleep improvement, attenuation of hyperarousal and anxiety [52–54], and reduced values in the Clinician Administered Posttraumatic Scale (CAPS [55];). Similarly, the results of open-label [56], populational [57], and double-blind placebo-controlled [56] studies have shown the benefits of using Δ^9^-tetrahydrocannabinol (THC), its synthetic version dronabinol or its analog nabilone to manage insomnia and nightmares in PTSD patients. However, contradictory results have also been reported, leading some authors to question the value of this *Cannabis*-based approach [58–61]. Differences in dose, route of administration, treatment regimen, level of THC tolerance, and current and past stress may account for the mixed findings above-mentioned [62]. Of note, the potential effects of THC/dronabinol or nabilone on aversive memory extinction and reconsolidation are under investigation. In contrast, it is still unknown whether cannabidiol (CBD), the main compound of *Cannabis* devoid of psychotomimetic effects, can impair the reconsolidation of aversive memories and facilitate their extinction in humans [63, 64], although its anxiolytic action has already been reported [65–68]. Similarly, associating THC with CBD could be therapeutically advantageous, but studies focusing on extinction or reconsolidation of aversive/traumatic memories are still incipient.

Based on the above, the present review aims to discuss the effects of THC, its synthetic version dronabinol, or its analog nabilone when administered alone and combined with CBD, on the extinction of aversive memories and anxiety, a common PTSD symptom, in healthy humans and PTSD patients.

## Methods

### Design

A qualitative systematic review of the research literature was carried out to identify relevant studies addressing the topics of our review.

### Study eligibility

#### Types of studies

Studies presenting primary data from controlled trials in healthy adults, anxious or PTSD patients that evaluated the effects of THC or its association with CBD on fear-related memories or anxiety responses, and published in English were included. The focus was on memory extinction/reconsolidation and anxiety-related responses.

#### Search strategy

Medline, PubMed, Cochrane Library and Central Register for Controlled Trials databases were searched using the keywords and MeSH terms [Δ^9^-tetrahydrocannabinol/THC or THC and cannabidiol/THC and CBD] and [Fear extinction/memory extinction/fear memory/memory reconsolidation or anxiety] for human studies and controlled trials published between 1974 and July 2020 in the English language. The reference lists of articles included and previous systematic reviews were checked for relevant publications. Primary studies presenting data from oral or smoked THC or THC plus CBD effects on fear-related memory or anxiety were identified.

#### Data abstraction

**Fig. 2:**
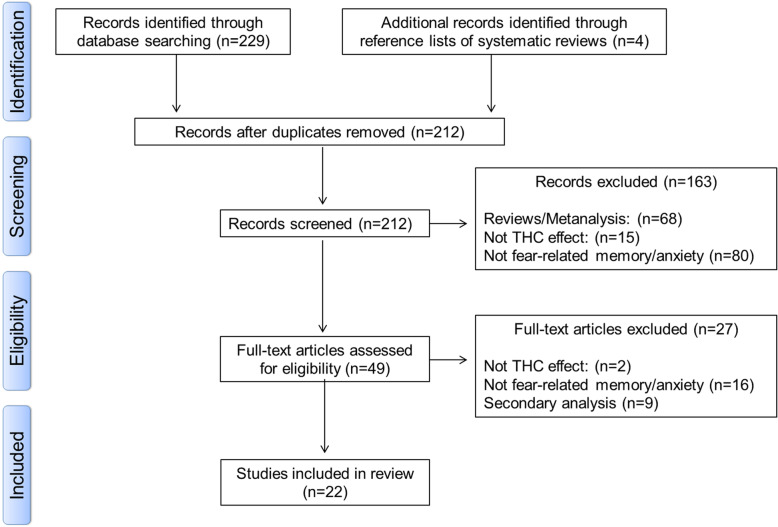
Flowchart of the study selection procedure

Abstracts were identified and independently analyzed by two reviewers. Three reviewers independently conducted data extraction and coding disagreements arising were discussed, and consensus coding applied. The following information was extracted for each study: age, gender, the health status of subjects, drug(s) used, doses and its management, protocols adopted, and the main results found. Figure 2 depicts the summary of the search process and the study exclusion criteria.

### Effect size calculation

Means, standard errors, and the number of subjects per group (“n”) were collected to calculate the effect size of treatment in the selected studies (raw data are summarized in Table S1 and S2). When the study presented only the standard deviation as the dispersion measure, the standard error was calculated using the following formula: standard deviation divided by the square root of “n”. When the means and the dispersion measure were depicted in the figure only, the measure tool from the Adobe Acrobat Reader® software was used to calculate them.

The effect size of behavioral results was calculated using the formula for Cohen’s *d* to reflect the mean-difference (± 95% confidence interval) between two groups. A *d* ≥ 0.5 and ≤ 0.8 was considered a medium effect size while a large one happened when *d* > 0.8 [69]. When the study did not present the dispersion measure or mention the “n” per group, the effect size was not calculated. The *d* values were all presented as positive values, and when the study presented repeated time points of the evaluated parameter, the *d* expressed in the text was the highest achieved.

## Results

### Effects of THC/dronabinol on aversive memory extinction or reconsolidation

Table 1 summarizes the main findings from the five double-blind studies investigating the effects of an acute administration of dronabinol on aversive memory extinction in healthy men and women. Neither studies nor clinical trials investigating the THC/dronabinol effects on aversive/traumatic memory reconsolidation were identified.

**Table 1:** The effects of THC or its synthetic analogs on fear extinction and emotional memory processing

Drug, dose, treatment regimen, and route of administration	Study’s design	Study’s details and outcome measures	Main results	Sex	Age in years	Health status	Cannabis consume	Reference
Dronabinol 7.5 mg, acute, 2 h prior to EL, v.o.	Randomized, double-blind, placebo-controlled	Image presentation associated with sound presentation and SCR	↓ SCR 24 h after EL	♂ ♀	21–45	Healthy	Non-users	[70]
Dronabinol 7.5 mg, acute, 2 h prior to EL, v.o.	Randomized, double-blind, placebo-controlled,	fMRI scanning, resting-state functional connectivity analysis, and SCR	↑ vmPFC and hippocampus activation during EMR; ↓ Amygdala activity during early extinction learning; ↔ SCR	♂ ♀	21–45	Healthy	Non-users	[71]
Dronabinol 7.5 mg, acute, 2 h prior to EL, v.o.	Randomized, double-blind, placebo-controlled,	fMRI scanning and Resting-state functional connectivity analysis	↓ Recovery of fear after 24 h; ↓ Amygdala-hippocampus static functional connectivity; ↑ Amygdala-vmPFC dynamic functional connectivity	♂ ♀	21–45	Healthy	Non-users	[72]
Dronabinol 7.5 mg, acute, 2 h prior to EL, v.o.	Randomized, double-blind, placebo-controlled,	fMRI scanning and SCR	↓ vmPFC and amygdala responses and ↑ functional coupling between the vmPFC, hippocampus and dACC 1 week later; ↓ SCR in first extinction trials; ↓ SCR in EMR 24 h later; ↔ 1 week after the EL	♂ ♀	21–45	Healthy	Non-users	[73]
Dronabinol 10 mg, acute, 2 h prior to EL, v.o.	Double-blind, placebo-controlled,	FPS and SCR	↓ SCR during EL ↔ FPS	♂ ♀	18–30	Healthy	Non-users	[74]

Legend: ↑ = increase; ↓ = reduction; ↔ no changes; ♂ = male; ♀ = female; v.o.: via oral; *EL* Extinction learning; *EMR* Extinction memory recall; *FPS* Fear-potentiated startle; *vmPFC* Ventromedial prefrontal cortex; *dACC* Dorsal anterior cingulate cortex; *fMRI* Functional magnetic resonance imaging; *SCR* Skin conductance response

Oral administration of 7.5 mg of dronabinol before fear extinction potentiated (*d* = 0.81) the extinction process in subjects submitted to a cued fear conditioning, as inferred by a reduced skin conductance response (SCR) during extinction recall 24 h later [70]. In a similar study [71], there were no dronabinol-induced SCR changes during extinction recall, even though it increased the activation of the vmPFC and hippocampus. In a subsequent study, dronabinol (7.5 mg/kg) attenuated the recovery of fear 24 h after extinction, and during a post-extinction resting period, the drug-treated subjects presented altered state brain dynamics (lowered amygdala-hippocampus static functional connectivity and increased amygdala-vmPFC dynamic functional connectivity) that were associated with a better extinction recall (*d* not calculated [72];).

Dronabinol effects on extinction have already been evaluated at a more remote time point. In the study by Hammoud et al., 2019 [73], subjects received 7.5 mg of this drug before extinction learning, and the retention test was performed both one and 7 days later. Dronabinol reduced the SCR during the extinction session (*d* = 0.55) and the retention test on day 1 (*d* = 0.55), but not on day 7. In the last case, however, the drug-treated group presented a significant increase in the functional coupling among the vmPFC, hippocampus, and dACC, which was associated with lower spontaneous recovery of fear. Moreover, healthy volunteers submitted to a neutral face presentation associated with an aversive sound and treated with 10 mg of dronabinol before extinguishing that association presented a transient reduction in SCR (*d* = 0.58), but unaltered fear-potentiated startle response [74].

In summary, in four of five studies, dronabinol induced significant changes in extinction-related autonomic or behavioral responses, with medium to large effect sizes (i.e., from 0.55 to 0.81). Therefore, this acute pharmacological intervention is associated with clinically relevant effects. There were no sex-dependent differences in dronabinol-induced facilitating effects on aversive memory extinction. However, such action will possibly require periodic associations of drug administration with extinction to be preserved. Noteworthy, the effects of repeated THC/dronabinol administration on aversive/traumatic memory extinction are still unknown. Despite the current knowledge about aversive memory reconsolidation and the well-documented role of the endocannabinoid system in this process in laboratory animals [75, 76], no human studies have addressed this question yet.

### Effects of THC/dronabinol plus CBD on aversive memory extinction or reconsolidation

No human studies or clinical trials investigating the effects of associating THC with CBD on aversive/traumatic memory extinction or reconsolidation were identified. However, they are foreseen since the combination of THC and CBD presents advantages in comparison with THC/dronabinol alone, such as fewer and less intense adverse effects and greater safety [77, 78]. Accordingly, in the studies evaluated, whereas 10–15 mg of THC often induce psychosis in either healthy or susceptible individuals, measured by Positive and Negative Psychotic Syndrome Scale (PANSS) general scores (*d* = from 0.84 to 2.52 [79–83];), 50 mg of THC associated with CBD in a dose ratio of ~ 1:1 (nabiximols, Sativex®) no longer produced that psychotomimetic effect [78, 84]. The antipsychotic effect of CBD, which can also counteract some other undesired effects related to CB1 receptor activation by THC, probably explains this pattern of results [85, 86]. However, some studies have indicated that CBD might not counteract the THC psychotic effect (for a review, please see [87, 88]).

### Effects of THC/dronabinol or its analog nabilone on anxiety-related responses

Table 2 summarizes the main findings from the 17 studies investigating the effects of THC/dronabinol or nabilone on anxiety in healthy humans and patients with PTSD or anxiety disorders.

**Table 2:** Effects of THC, dronabinol, nabilone or THC and CBD on anxiety-related responses

Drug(s), dose(s), treatment regimen, and route of administration	Study’s design	Main results	Sex	Age in years	Health status	Cannabis consume	Reference
THC 10 mg, acute, v.o.	Randomized, double-blind, placebo-controlled	↑ anxiety on VAMS	♂	18–42	Healthy	Non-users	[79]
THC 10 mg, acute, v.o.	Pseudorandomized, double-blind, placebo-controlled	THC ↑ anxiety on STAI and VAMS; ↑ SCR and left amygdala activity viewing fearful faces	♂	20–42	Healthy	Non-users	[80]
THC 10 mg, acute, v.o.	Pseudorandomized, double-blind, placebo-controlled	↑ anxiety on STAI; ↑ CB1 activation in right amygdala while viewing fearful faces	♂	23.79 ± 4.45	Healthy	Non-users	[81]
THC 10 mg, acute, v.o.	Randomized, double-blind, placebo-controlled	↑ anxiety on STAI in both groups; ↑ left fusiform gyrus, and ↓ left precuneus, cuneus and posterior cingulate activity in Cannabis users	♂	26 ± 5.6	Healthy	Users and non-users	[82]
THC 10 mg, acute, v.o.	Randomized, double-blind, placebo-controlled	THC ↑ anxiety on STAI, ↑ SCR, ↑ activity of right inferior, right superior and left medial frontal gyrus and ↓ left precuneus during the processing of fearful faces	♂	18–35	Healthy	Non-users	[83]
Nabilone 2.0 mg, acute, v.o.	Randomized, double-blind, placebo-controlled	↔ anxiety during a non-traumatic psychological stress	♂	18–30	Healthy	Non-users	[89]
Dronabinol 7.5 mg, acute, v.o.	Randomized, double-blind, placebo-controlled	↔ anxiety; ↓ amygdala reactivity viewing fearful faces	♂ ♀	18–28	Healthy	Non-users	[90]
Dronabinol 7.5 or 12.5 mg, acute, v.o.	Randomized, double-blind, placebo-controlled	7.5 mg ↓ stress reactivity on VAS; 12.5 mg ↑ stress reactivity and anxiety on POMS	♂ ♀	18–40	Healthy	Non-users	[91]
THC 0.5 mg/kg + CBD 1.0 mg/kg, acute, v.o.	Double-blind, placebo-controlled	THC ↑ anxiety on STAI; CBD ↓ the THC effects	♂ ♀	20–38	Healthy	Non-users	[92]
THC 30 mg + CBD 15, 30 or 60 mg, acute, v.o.	Double-blind, placebo-controlled	THC ↑ anxiety; CBD 30 or 60 mg ↓ most of the effects of THC	♂	adults	Healthy	Non-users	[93]
THC ~ 14 mg, acute, via inhalation (smoked)	Double-blind	↔ anxiety on MAACL-T; ↓ SCR and forearm blood flow	♂	18–29	Healthy	Users	[94]
THC 1.8% or 3.6% + CBD 0.2% or 1.0%, acute; smoked	Randomized, double-blind, placebo-controlled	THC 3.6% ↑ anxiety; THC 3.6% + CBD 1.0% ↔ anxiety; THC 3.6% + CBD 0.2% ↑ anxiety; THC 1.8% ↔ anxiety; THC 1.8% + CBD 1.0% ↑ anxiety; THC 1.8% + CBD 0.2% ↔ anxiety	♂ ♀	21–45	Healthy	Users	[95]
THC 5.0 or 15 mg; “low” Sativex® (THC 5.4 mg + CBD 5.0 mg CBD); or “high” Sativex® (THC 16.2 mg + CBD 15 mg); acute, v.o. and oromucosal	Randomized, double-blind, placebo-controlled	↑ anxiety with THC 5 or 15 mg; ↔ anxiety with “low” Sativex® ↑ anxiety with “high” Sativex®	♂ ♀	18–45	Healthy	Users	[96]
Nabilone 1.0, 2.0, 4.0 or 5.0 mg, acute, v.o.	Single-blind, placebo-controlled	1.0 and 2.0 mg ↓ anxiety on POMS	♂ ♀	adults	Anxious	Non-users	[97]
Phase 1: nabilone 1.0–10 mg; Phase 2: nabilone 1.0 mg; 28 days t.i.d. administration	Phase 1: Open-label Phase 2: Double-blind, placebo-controlled	↓ anxiety	♂ ♀	19–41	Anxious	Non-users	[98]
THC 5.0 mg, 21 days t.i.d. administration, v.o.	Open-label	↓ anxiety and improved global symptoms	♂ ♀	adults	PTSD	Non-users	[99]
Dronabinol 7.5 mg, acute, v.o.	Randomized, double-blind, placebo-controlled	THC ↓ amygdala activation during threatening faces on PTSD patients and during non-threatening faces on healthy controls; ↑ mPFC/rACC-amygdala functional connectivity ↔ anxiety on STAI	♂ ♀	20–45	PTSD, trauma exposed, and healthy controls	Non-users	[100]

Legend: ↑ = increase; ↓ = reduction; ↔ = no change; ♂ = male; ♀ = female; v.o. = oral route; *t.i.d.* Twice in day; *mPFC* Medial prefrontal cortex; *rACC* Rostral anterior cingulate cortex; *STAI* STATE Trait Anxiety Inventory; *VAMS* Visual Analogue Mood Scale; *VAS* Visual Analogue Scale; *POMS* Profile of Mood State; *SCR* Skin conductance response

Oral administration of 2.0 mg of nabilone to healthy subjects with no history of *Cannabis* consumption produced no changes in the anxiety state measured by the Hopkins Symptom Checklist Scores [89]. In contrast, 7.5 mg of THC decreased both stress reactivity, measured by the Visual Analog Scales (VAS) in the Trier Social Stress Test (*d* = 0.82), and amygdala activation when viewing fearful faces [90, 91]. THC (7.5 mg) also increased subjective reports of “high”, but neither impairments in task performance nor changes in anxiety, sedation, and arousal, as measured by the State-Trait Anxiety Inventory (STAI) and the Profile of Mood State (POMS), were reported [90, 91]. When administered orally at doses ≥10 mg, THC was reported to increase the anxiety state, as measured by STAI (*d* = from 0.93 to 2.52 [79–83, 92];), Visual Analogue Mood Scale (VAMS; *d* = 0.63 [79];), POMS (*d* = 1.32 [91];), subjective reports [91] or SCR during presentation of neutral and fearful faces (*d* = from 0.92 to 1.42 [81, 83];). This anxiogenic THC action has been associated with increased activation of CB1 receptors in the right amygdala [80, 81]. It was also accompanied by increased activation in frontal and parietal areas [83].

In healthy subjects with a history of *Cannabis* consumption, smoking a cigarette containing 1.8% (~ 15 mg) of THC produced no changes in the anxiety state [94, 95]. In contrast, either smoking 3.6% of THC or its oral intake of 5.0 to 15 mg increased the anxiety state measured by STAI and VAS (*d* not calculated [82, 95, 96];). Similarly, 10 mg of THC given orally increased the anxiety state measured by STAI in both *Cannabis* users and non-users (*d* not calculated), but the induction of psychotic symptoms was less intense in the former group [82]. Of note, the brain areas influenced by THC in users and non-users of *Cannabis* differed significantly [82, 83]. The explanation for the varying pattern of results is still under debate.

In anxious patients, oral administration of 1.0 or 2.0 mg of nabilone attenuated (d not calculated) the anxiety response measured using POMS, an effect accompanied by increased heart rate. At doses of 4.0 and 5.0 mg, nabilone produced orthostatic hypotension without further anxiety reduction (*d* not calculated [97];). Reduced anxiety was also reported in a study (*d* not calculated; 100) divided into two phases: the first being open-label with five patients receiving nabilone on an escalating dose regimen starting with 1.0 mg and not exceeding 10 mg, for 28 days; and the second being double-blind, placebo-controlled with 20 patients receiving nabilone 1.0 mg twice a day for 28 days. The adverse effects reported were drowsiness and dry mouth and eyes [98].

In an open-label study using PTSD patients [99], orally administering 5.0 mg of THC twice a day for 21 days improved their anxiety (*d* = 1.16) and global state. A recent double-blind study [100] investigated PTSD patients, trauma-exposed individuals, and healthy controls acutely treated with THC (7.5 mg). No significant changes were found in the anxiety state, but THC lowered threat-related amygdala reactivity, increased mPFC activation during the threat, and increased mPFC-amygdala functional coupling in PTSD patients [100].

In summary, in most of the studies above described, THC/dronabinol or nabilone induced statistically significant effects on anxiety-related responses, with medium to large effect sizes (i.e., from 0.63 to 2.52). However, the outcome (anxiolytic or anxiogenic effect) depends on the dose, regimen of treatment, and psychiatric status. When considered, there were no sex-related differences in drug-induced effects on anxiety.

### Effects of THC/dronabinol plus CBD on anxiety-related responses

As detailed in Table 2, four double-blind studies evaluated the effects of associating THC with CBD on anxiety in healthy users and non-users of *Cannabis* [92, 93, 95, 96]. CBD was able to attenuate the anxiogenic effect of THC when given in a THC:CBD dose ratio of 1:1 or 1:2 (*d* = from 4.33 to 4.59), but not 1:0.5 or 1:0.33 [92, 93, 95]. Of note, the THC-induced increase in heart rate frequency was reduced in the presence of CBD (*d* = 3.41; THC:CBD dose ratio of 1:2 and d = 1.90 THC:CBD dose ratio of 1:1 [91];). Furthermore, the use of a THC:CBD dose ratio of ~ 1:1 high Sativex® (THC 16.2 mg + CBD 15 mg), but not low Sativex® (THC 5.4 mg + CBD 5 mg) induced an increase in anxiety state (*d* not calculated [96];). Besides, High Sativex induced higher “feeling anxious” parameters (measured by VAS) than Low Sativex (*d* = 0.58 [96];). Smoking a cigarette containing THC 3.6% (~ 30 mg) and CBD 1.0% (~ 8.5 mg) was associated with less anxiety (*d* not calculated) than the one containing only THC 3.6%. In contrast, smoking a cigarette with THC 1.8% (~ 15 mg) alone produced no changes in anxiety, but it was increased when the same THC dose was associated with CBD 1.0% [95].

In summary, CBD attenuates or even prevents the anxiogenic action produced by higher THC doses when given in a dose similar to or higher than that of THC. This outcome, however, depends on their absolute quantity and route of administration. Importantly, no study has already evaluated the effect of chronic use of CBD and THC on anxiety. When considered, there were no sex-related differences in THC-induced effects on anxiety, as well as in the potential counteracting CBD action.

## Discussion

The facilitating effects of dronabinol (7.5–10 mg) in humans undergoing aversive memory extinction agree with laboratory animal data showing that the activation of CB1 receptors plays a crucial role in fear memory extinction [101–104]. For instance, THC and CBD treatment facilitates extinction acquisition and recall, respectively [101, 105]. The action of dronabinol above mentioned is also in line with human studies investigating the THC effects on procedures involving threat perception recognition, such as the emotional face-matching task, the facial emotion recognition, and the recognition of emotional pictures. At a dose range from 7.5 to 15 mg, it reduced the threat perception, the recognition of emotional pictures, and fear and anger faces [106–109]. THC also enhanced the functional connectivity of specific brain areas, such as the amygdala with both rostral anterior cingulate and medial prefrontal cortices, during the emotional face-matching task and extinction recall [106]. This pattern of results suggests that THC/dronabinol interferes with aversive memory processing and its extinction. Of note, impairments in recognition of non-emotional pictures were found after administering 15 mg of THC [110], indicating that high THC doses can affect neutral memory processing.

The studies focusing on aversive memory extinction in healthy humans and laboratory animals are compelling. However, future studies are guaranteed to examine the effects of acute and chronic THC/dronabinol administration on the extinction of traumatic memories. Currently, there are two double-blind and placebo-controlled study investigating THC effects on extinction learning in PTSD patients (ClinicalTrials.gov Identifier: NCT03008005; ClinicalTrials.gov Identifier: NCT04080427). The preliminary results indicate that PTSD patients treated with THC (7.5 mg) exhibited better recall of the extinction of a cued aversive memory, an effect accompanied by increased hippocampal activation [111]. Although this result agrees with that reported in healthy subjects, the traumatic memory was not evaluated and, thus, whether and how THC can attenuate its expression is still unknown. Moreover, as early mentioned, the extinction’s ability to suppress the original aversive/traumatic memory is thought to be temporary. Based on this, it would be interesting to investigate whether THC/dronabinol can hinder or even prevent extinction-related features that limit its clinical usefulness, such as renewal, reinstatement, and spontaneous recovery over time. Furthermore, some studies report that the long-term use of *Cannabis* by individuals with PTSD is associated with worse clinical outcomes [61, 112], however, investigating the effects of THC either alone or combined with CBD on PTSD symptoms at clinical settings cannot be directly compared with smoking cannabis in an uncontrolled environment, and possibly in a recreational manner. For more details, see 113.

No sex-dependent differences in THC-induced effects on extinction were identified. Women who have PTSD present more CB1 receptor expression and lower hair concentrations of PEA, OEA, and SEA, than men [40, 42]. The CB1 receptor density has been inversely correlated with anandamide and other endocannabinoid levels, implying that men had a high concentration of endogenous ligands, which might be a factor contributing not only to the increased risk of developing stress-related disorders but also to symptom severity in women [40]. In blood samples, the availability of CB1 receptors is also higher in women than men [113]. In rodents, gonadal hormones (e.g., estradiol) regulate the CB1 receptor density [114], transcription [115], and signal transduction [116], which ultimately influences the content of endocannabinoids [117] and their CB1 receptor affinity [118]. Estradiol can facilitate the fear extinction process [119, 120]. Moreover, 4 out of 5 of the studies [70–73] have reported that women that were not taking hormonal contraceptives were tested during their follicular phase only to avoid between-group effects of sex hormones. When female participants were included in the studies, they were performed about 1 week before menses onset (based on self-reports of last period and cycle length), when the estrogen level is low. This is likely a factor contributing to the lack of significant sex-dependent differences in THC effects on extinction. Of potential relevance to the present discussion are results showing that a deficient conversion of progesterone to its neuroactive metabolite allopregnanolone, which facilitates GABA action on GABA_A_ receptors, in women with PTSD was associated with resistance to extinguish the fear memory [121, 122]. Moreover, pregnanolone, a precursor of progesterone, is a negative allosteric modulator of CB1 receptors [123]. How the interplay between steroid hormones and the endocannabinoid system contributes to fear extinction is currently under investigation. Altogether, studies support sex-dependent fluctuations in some endocannabinoid system constituents involved in extinction processing. However, animal studies have been traditionally performed almost exclusively in males. Similarly, human studies do not frequently compare THC effects on men and women. In any case, complementary analyses are guaranteed.

No human studies investigating THC effects on aversive memory reconsolidation were identified. The number of animal studies focusing on this question is still scarce, although their findings are promising. In a dose range from 0.3 to 10 mg/kg, the acute and systemic treatment with THC impaired the reconsolidation of specific and recent contextual fear memories in adult male rats, an effect dependent on prelimbic cortex CB1 receptor activation [124]. The acute treatment with 5.0 (but not 50) mg/kg of THC similarly impaired the reconsolidation of a recent cued fear memory in male rats [125]. Future studies should address whether THC can impair the reconsolidation of more remote and generalized aversive memories in both male and female animals. Noteworthy, mixed results have been reported with other drugs (e.g., propranolol) targeting aversive memory reconsolidation in rodents versus healthy humans [30, 33, 126–130], which suggests the existence of some boundary conditions, such as the strength, age, and specificity of the memory to be pharmacologically “fine-tuned”. Fortunately, animal findings indicate that the use of behavioral and pharmacological strategies can surmount these constraining factors [29, 131].

THC has been pointed out as a therapeutic cannabinoid for several brain diseases [132]. However, it is also the main responsible for the recreational use of *Cannabis* and its psychotomimetic effect, restricting *Cannabis* therapeutic use. Besides, some THC effects are commonly biphasic and dose-dependent [133]: low doses have potential therapeutic value in cognitive and anxiety disorders while high doses cause harmful effects, and are related to the reported side effects resulting from direct activation of CB1 receptors [51, 131]. This supraphysiological action can lead to a rapid down-regulation of these receptors [134, 135], potentially resulting in tolerance and addiction [136, 137]. CBD, on the other hand, has a safer profile because it is not a psychotic-precipitating substance, and its effects are mediated via indirect CB1 receptor activation and non-cannabinoid mechanisms as well [138, 139].

No human studies have yet investigated the effects of combining THC with CBD on aversive/traumatic memory extinction or reconsolidation. As early mentioned, however, this association could be advantageous. Accordingly, administering both THC and CBD in a sub-effective dose impaired either contextual or cued fear memory reconsolidation in adult male rats [124, 125]. In both cases, the THC:CBD dose ratio was 1:10, and combining THC with CBD allowed the use of 3-fold lower doses of them in the study by Stern et al. [124].

Regarding the effects of THC, dronabinol or nabilone on anxiety in subjects with no history of *Cannabis* use, orally taken doses ≥10 mg increased the anxiety level (and often produced a psychotic effect) while lower doses produced either no changes or anxiety reduction. Although the anxiogenic and psychotic effects of THC could have clinic impact (their effect sizes are as large as the ones calculated here for extinction facilitation and anxiety reduction), the dose range in which the beneficial and detrimental THC effects predominates is not necessarily the same. Therefore, its therapeutic value depends on the dose. Another aspect to keep in mind is the treatment regimen. Repeated administration of 1.0 or 5.0 mg of THC reduced anxious symptoms in anxious or PTSD patients, but acute treatment produced minor or no effects. In *Cannabis* users, the effects on anxiety are more variable because of the influence of the previous emotional state of individuals, how long have they used *Cannabis*, and when in life they started its use. Of note, inhalation is an alternative route to deliver THC. THC bioavailability after oral administration is around 10% [140], and after smoking, 25% of it reaches systemic circulation [141]. Based on that, a given smoked dose of THC will produce higher blood concentration than orally and, thus, the anxiogenic effect would be more frequent. However, in both studies in which this question was addressed indirectly, the subjects were previous *Cannabis* users, possibly having some degree of tolerance to the anxiogenic effect of THC. Besides, chronic users of *Cannabis* present altered anxiety levels relative to non-users [142]. Thus, the downregulation of CB1 receptors and differences in anxiety’s baseline could also have influenced THC effects in *Cannabis* users.

The biphasic effects of THC on anxiety have also been shown in laboratory animals. Low doses of THC (e.g., 0.3 mg/kg) did not alter the anxiety response in rodents tested in the elevated plus-maze, but doses between 1.0 and 10 mg/kg produced an anxiogenic-like effect [143]. In another study, THC doses ranging from 0.075 to 1.5 mg/kg produced an anxiolytic-like effect [144]. Interestingly, the anxiolytic and anxiogenic effects of THC can arise from different brain areas: infusing low doses into the ventral hippocampus or medial prefrontal cortex induced an anxiolytic-like response while higher doses had no effect or even produced an anxiogenic-like effect. In contrast, the same low THC dose infused into the basolateral amygdala increased the anxiety response, but higher doses were ineffective. Both effects relied on the activation of CB1 receptors [145], which are located on glutamatergic and GABAergic neurons. It is hypothesized that THC reduces glutamate levels in low doses and raises glutamate levels in high doses (the latter action is associated with inhibition of GABA-releasing neurons [146];). Furthermore, Bedse et al. [147] demonstrated in mice that the 2-AG depletion-induced anxiety-like behavior after stress exposure was counteracted by the administration of 0.25 mg/kg THC. Overall, these findings confirm the bidirectional effects of THC on anxiety, despite the varying dose range associated with anxiolytic and anxiogenic effects [148]. Of note, at doses ranging from 3.0 to 10 mg/kg in rats, and from 10 to 20 mg/kg in mice, THC also produced sedative-like effects [143]. The neural basis underlying THC effects on anxiety and sedation are under investigation.

Associating CBD with THC could be advantageous as CBD can minimize or even counteract some adverse effects of THC, such as the anxiogenic and psychotic effects. Oral administration of THC plus CBD in a dose ratio 1:2 attenuated the THC-associated anxiogenic effect. When using a similar THC:CBD ratio, the THC-associated anxiogenic effect was no longer observed with the use of low, but not high, doses of these drugs, a result that also depended on the previous *Cannabis* use history of participants. In humans, oral pretreatment with CBD 600 mg attenuated the psychotic symptoms induced by THC 10 mg [80]. Similarly, 400 mg, but not 4.0 mg, of CBD vaporized attenuated the intoxication produced by THC 8.0 mg [149]. Preclinical studies with animals also demonstrate that co-administration of THC:CBD in 1:5 and 1:10, but not 1:1, dose ratio can counteract the THC-induced anxiogenic-like effects and impairments in social interaction [150, 151]. Based on the above, the combined strategy seems to be as complex as using THC alone.

There is evidence suggesting that CBD interferes with the pharmacokinetics of THC. Indeed, CBD can inhibit the hepatic metabolism of THC [152, 153]. As a result, co-administering an equal dose of CBD doubled the brain THC amount, 30 min later, in adolescent male rats and, thus, this association produced an anxiogenic-like effect [154]. Similarly, female mice receiving a THC:CBD dose ratio of 1:2 presented an anxiogenic-like effect [155]. However, studies using proportionally more CBD than THC and performed in adult rodents and monkeys found a reduction in THC anxiogenic action [156–158], indicating that the hepatic biotransformation of THC may vary according to the animal’s age. In men, independent of dose ratio, the oral co-administration of CBD with THC did not alter the plasmatic THC concentration [80, 96, 159]; in women, the intake of a THC:CBD dose ratio of 1:0.5 induced a tendency of increasing both THC and its active metabolite (11-OH-THC) in the plasma, suggesting potential sex-dependent differences in THC and CBD metabolism [159]. Altogether, CBD can interfere with the pharmacokinetics of THC, but the dose, animal species, sex, and proportion of these drugs influence if CBD will potentiate or antagonize THC effects.

## Conclusions

THC, dronabinol or nabilone could help with hyperarousal symptoms, insomnia, anxiety, and extinction deficits related to PTSD [51]. Indeed, despite the limited number of published studies, available data suggest that low doses of THC potentiate fear memory extinction in healthy volunteers and reduce anxiety responses in anxious and PTSD patients without inducing a psychotic effect. High doses of THC, however, do not facilitate fear memory extinction and are related to clinically relevant anxiogenic and psychotic effects in healthy volunteers. Overall, laboratory animal data corroborate human findings.

There is a lack of studies with PTSD patients using THC alone and associated with CBD focusing on aversive memory extinction and reconsolidation. Further, most studies evaluated the acute effects of THC or THC plus CBD. Therefore, it is unknown whether chronic treatment is still advantageous. Besides, some studies do not address potential sex-dependent differences in THC-induced effects, which would provide further information on whether or not it is a potential issue in humans. Animal data have shown the detrimental effects of THC following high doses. Based on that, human studies have selected an appropriate dose range of THC and, thus, neither worsening of PTSD symptoms nor strengthening of aversive memories after the use of THC has been reported. Few studies have investigated the effects of associating THC with CBD in varying dose ratios yet. Altogether, the findings encourage future controlled studies evaluating the effects of low doses of THC to attenuate aversive/traumatic memory expression in PTSD patients.

## Supplementary information

**Additional file 1 Supplementary Table 1.** Raw data used for calculate the effect sizes of behavioral parameters from studies detailed in Table 1.

**Additional file 2 Supplementary Table 2.** Raw data used for calculate the effect sizes of behavioral and autonomic parameters from studies detailed in Table 2.

## Data Availability

All data generated or analyzed during this study are included in this published article [and its supplementary information files].

## Abbreviations

2-AG: 2-Arachidonoylglycerol
Caps: Clinician administered posttraumatic scale
CB1: Cannabinoid type 1 receptor
CBD: Cannabidiol
dACC: Dorsal anterior cingulate cortex
FAAH: Fatty acid amide hydrolase
mPFC: Medial prefrontal cortex
OEA: Oleoylethanolamide
PANSS: Positive and negative psychotic syndrome scale
PEA: Palmitoylethanolamide
POMS: Profile of mood state
PTSD: Posttraumatic stress disorder
SEA: Stearoylethanolamide
SCR: Skin conductance response
SSRIs: Selective serotonin reuptake inhibitors
STAI: State trait anxiety inventory
THC: Δ^9^-tetrahydrocannabinol
VAS: Visual analog scale
vmPFC: Ventromedial prefrontal cortex
