# Supplementary material for: Effects of ∆9-tetrahydrocannabinol on aversive memories and anxiety: a review from human studies
Source: BMC Psychiatry. 2020 Aug 26;20:420. doi: 10.1186/s12888-020-02813-8 (PMC7448997; doi:10.1186/s12888-020-02813-8)
Supplement: Supplementary file 1 — Additional file 1 Supplementary Table 1. Raw data used for calculate the effect sizes of behavioral parameters from studies detailed in Table 1. [file 12888_2020_2813_MOESM1_ESM.docx]

Supplementary table 1. Raw data used for calculate the effect sizes of behavioral parameters from studies detailed in Table 1.

| **Control x drug** | **Effect observed** | **Control** | | | **Drug** | | | **Cohen’s *d* effect size [± 95% C.I.]** | **Reference** |
| --- | --- | --- | --- | --- | --- | --- | --- | --- | --- |
|  |  | **Mean** | **SEM** | ***n*** | **Mean** | **SEM** | ***n*** |  |  |
| Dronabinol (THC)  7.5mg x Placebo | ↓ SCR | 0.136 | 0.102 | 14 | -0.14 | 0.082 | 15 | 0.81 [0.05-1.57] | (72) Figure 3. SCR during extinction. |
| Dronabinol (THC)  7.5 mg x Placebo | ↓ SCR in first extinction trials | 0.21 | 0.06 | 20 | 0.09 | 0.03 | 20 | 0.55 [0.08-1.18] | (75) Figure 2A. SCR in the 1-5 extinction trials. |
| Dronabinol (THC)  7.5 mg x Placebo | ↓ SCR in extinction recall | 0.23 | 0.08 | 20 | 0.09 | 0.03 | 20 | 0.55 [0.08-1.19] | (75) Figure 3A. SCR during extinction retention test. |
| Dronabinol (THC)  10 mg x Placebo | ↓ SCR | 0.42 | 0.08 | 18 | 0.25 | 0.06 | 18 | 0.58 [0.09-1.25] | (76) Figure 4A. SCR response. |

Legend: ↓ = reduction; SCR = skin conductance response.
