# Supplementary material for: Effects of ∆9-tetrahydrocannabinol on aversive memories and anxiety: a review from human studies
Source: BMC Psychiatry. 2020 Aug 26;20:420. doi: 10.1186/s12888-020-02813-8 (PMC7448997; doi:10.1186/s12888-020-02813-8)
Supplement: Supplementary file 2 — Additional file 2 Supplementary Table 2. Raw data used for calculate the effect sizes of behavioral and autonomic parameters from studies detailed in Table 2. [file 12888_2020_2813_MOESM2_ESM.docx]

Supplementary table 2. Raw data used for calculate the effect sizes of behavioral and autonomic parameters from studies detailed in Table 2.

| **Control x drug** | **Effect observed** | **Time or condition of test** | **Control** | | | **Drug** | | | **Cohen’s *d* effect size [± 95% C.I.]** | **Reference** |
| --- | --- | --- | --- | --- | --- | --- | --- | --- | --- | --- |
|  |  |  | **Mean** | **SEM** | ***n*** | **Mean** | **SEM** | ***n*** |  |  |
| THC x Placebo | ↑ STAI state | 2 h  3 h | -0.63  -1.31 | 1.72  1.42 | 16 | 7.28  7.02 | 1.76  3.1 | 16 | 1.17 [0.42 - 1.92]*  0.89 [0.16 - 1.62] | (81) Fig 1. Two and 3 h after treatment. |
| THC x Placebo | ↑ VAMS Anxiety | 2 h  3 h | -0.42  -0.56 | 1.8  1.38 | 16 | 3.63  3.16 | 1.49  2.11 | 16 | 0.63 [0.08 - 1.34]*  0.54 [0.17 - 1.24] | (81) Fig 2. Two and 3 h after treatment. |
| THC x Placebo | ↑ PANSS general score | 2 h  3 h | 0.00  -0.65 | 0.48  0.3 | 16 | 5.62  2.65 | 1.59  1.17 | 16 | 1.24 [0.48 - 1.99]*  1.00 [0.26 - 1.73] | (81) Fig 4. Two and 3 h after treatment. |
| THC x Placebo | ↑ PANSS score | 2 h  3 h | 7.07  7.00 | 0.05  0.03 | 15 | 9.57  7.71 | 1.1  0.18 | 15 | 0.86 [0.11 - 1.61]  1.47 [0.66 - 2.28]* | (82) Fig 3. Two and 3 h after treatment. |
| THC x Placebo | ↑ STAI Score | 2 h  3 h | 12.76  12.08 | 2.4  2.17 | 15 | 22.35  22.03 | 2.76  2.08 | 15 | 0.99 [0.23 - 1.75  1.25 [0.47 - 2.03]* | (82) Fig 2. Two and 3 h after treatment. |
| THC x Placebo | ↑ STAI state | 2 h  3 h | 12.69  11.70 | 2.14  2.04 | 14 | 21.25  18.01 | 2.90  2.95 | 14 | 0.93 [0.15 - 1.71]*  0.69 [0.07 - 1.45] | (83) Fig 3. Two and 3 h after treatment. |
| THC x Placebo | ↑ PANSS total score | 2 h  3 h | 32.86  30.77 | 1.33  1.97 | 14 | 41.27  40.47 | 2.63  3.29 | 14 | 1.12 [0.32 - 1.92]*  0.99 [0.21 - 1.78] | (83) Fig 3. Two and 3 h after treatment. |
| THC x Placebo | ↑ Amplitude of SCR fluctuations | Neutral  Fearful | 0.14  0.21 | 0.05  0.04 | 14 | 0.51  0.35 | 0.15  0.09 | 14 | 0.92 [0.14 - 1.70]*  0.56 [0.20 - 1.31] | (83). Fig 4. Neutral and fearful faces. |
| THC x Placebo | ↑ in cannabis users  PANSS | 2 h  3 h | 33.18  31.35 | 1.29  0.58 | 12 | 38.65  35.82 | 2.21  1.39 | 12 | 0.92 [0.08 - 1.76]  1.26 [0.39 - 2.14]* | (84) Fig 1. Positive and Negative Syndrome Scale |
| THC x Placebo | ↑ in non-users  PANSS | 1 h  2 h  3 h | 31.11  31.30  30.78 | 0.43  0.53  0.33 | 12 | 36.54  45.10  43.32 | 1.77  3.79  4.47 | 12 | 1.27 [0.39 - 2.15]  1.54 [0.63 - 2.45]*  1.19 [0.32 - 2.06] | (84) Fig 1. Positive and Negative Syndrome Scale |
| THC x Placebo | ↑ PANSS | 1 h  2 h | 7.23  7.11 | 0.26  0.001 | 7 | 8.35  9.66 | 0.05  1.16 | 8 | 2.52 [1.16 - 3.88]*  1.13 [0.04 - 2.22] | (85) Fig 1A. Positive Syndrome Score |
| THC x Placebo | ↑ anxiety STAI score | 1 h  2 h | 14.59  12.87 | 0.31  2.13 | 7 | 19.98  23.75 | 0.29  1.25 | 8 | 0.71 [0.34 - 1.75]  2.52 [1.17 - 3.89]* | (85) Fig 1B. Positive Syndrome Score |
| THC x Placebo | ↑ No. of SCR fluctuations | Mildly fearful  Intensely fearful | 1.69  2.79 | 0.33  0.68 | 7 | 3.42  6.27 | 0.56  1.24 | 8 | 1.42 [0.28 - 2.55]*  1.30 [0.19 - 2.42] | (85) Fig 3. No. of SCR fluctuations |
| Nabilone x Placebo | ↔ Anxiety | 30 min | 2.00 | 0.75 | 12 | 3.4 | 0.87 | 12 | 0.52 [0.29 - 1.33] | (89) Table 1. Mean decreases in Hopkins Symptom Checklist Scores |
| THC 7.5 x Placebo | ↓ Subjective Distress | During TSST | 183.1 | 29.0 | 13 | 102.1 | 26.9 | 14 | 0.82 [0.03 - 1.61]* | (91) Table 4. Subjective distress score. |
| THC 12.5 x Placebo | ↑ POMS anxiety score | Pre to Post treatment changes | -0.06 | 0.04 | 13 | 0.29 | 0.09 | 15 | 1.32 [0.50 - 2.14]* | (91) Table 3. POMS score. |
| THC, CBD or THC and CBD x Placebo | ↑ STAI State | THC  CBD  THC and CBD | 0.125 | Not showed | 8 | 15.938  0.125  8.813 | Not showed | 8 | Not calculated | (92) Table 3. STAI State. |
| THC 30 mg x Placebo | ↑ Pulse rate | 30  50  70  90  110  150  170 | 96.6  98.2  92.4  91.8  88.8  86.8  87.0 | 2.8  3.7  2.4  3.9  3.5  2.7  4.7 | 5 | 123.6  135.2  133.4  130.2  119.4  110.2  109.4 | 7.6  8.7  5.5  8.2  6.8  4.4  3.6 | 5 | 2.36 [0.74 - 3.97]  2.77 [1.03 - 4.50]  4.83 [2.38 - 7.28]  2.99 [1.19 - 4.79]  2.36 [0.74 - 3.97]  3.20 [1.33 - 5.08]  2.67 [0.97 - 4.38] | (93) Table 1. Pulse rate (30 to 170 min) |
| THC 30 + CBD 30 x THC 30 | ↓ Pulse rate | 30  50  70  90  110  150  170 | 123.6  135.2  133.4  130.2  119.4  110.2  109.4 | 7.6  8.7  5.5  8.2  6.8  4.4  3.6 | 5 | 102.8  111.8  109.0  105.4  102.4  103.6  99.6 | 5.2  5.8  7.2  6.2  6.6  13.1  10.0 | 5 | 1.60 [0.17 - 3.02]  1.58 [0.16 - 3.00]  1.90 [0.41 - 3.40]*  1.71 [0.26 - 3.15]  1.27 [0.09 - 2.63]  0.34 [0.91 - 1.59]  0.65 [0.62 - 1.92] | (93) Table 1. Pulse rate (30 to 170 min) |
| THC 30 + CBD 60 x THC 30 | ↓ Pulse rate | 30  50  70  90  110  150  170 | 123.6  135.2  133.4  130.2  119.4  110.2  109.4 | 7.6  8.7  5.5  8.2  6.8  4.4  3.6 | 5 | 100.2  106.5  100.6  100.0  100.2  95.8  95.6 | 4.1  4.4  4.0  4.2  4.5  3.6  2.8 | 5 | 1.91 [0.42 - 3.41]  2.08 [0.54 - 3.62]  3.41 [1.47 - 5.35]*  2.32 [0.71 - 3.92]  1.66 [0.23 - 3.10]  1.79 [0.32 - 3.26]  2.14 [0.58 - 3.69] | (93) Table 1. Pulse rate (30 to 170 min) |
| THC 30 x Placebo | ↓ Time production task | T3  T4  T5  T6  T7  T8 | 58.3  59.6  59.4  59.6  57.8  51.0 | 1.0  0.6  0.7  0.7  1.4  2.7 | 5 | 33.6  40.2  39.6  49.2  39.9  51.0 | 2.1  2.6  2.1  3.3  2.5  2.7 | 5 | 7.51 [3.99 - 11.03]  5.14 [2.57 - 7.71]  6.32 [3.29 - 9.36]  2.18 [0.61 - 3.74]  4.42 [2.12 - 6.72]  2.27 [0.68 - 3.87] | (93) Table 2. Time production task (T3 to T8) |
| THC 30 + CBD 30 x THC 30 | ↑ Time production task | T3  T4  T5  T6  T7  T8 | 33.6  40.2  39,6  49.2  39.9  51.0 | 2.1  2.6  2.1  3.3  2.5  2.7 | 5 | 51.8  56.6  50.9  55.8  45.8  56.7 | 2.1  2.0  1.5  1.8  1.7  2.3 | 5 | 4.33 [2.06 - 6.60]*  3.53 [1.55 - 5.52]  3.10 [1.26 - 4.93]  1.24 [0.11 - 2.59]  1.38 [0.00 - 2.76]  1.14 [0.20 - 2.47] | (93) Table 2. Time production task (T3 to T8) |
| THC 30 + CBD 60 x THC 30 | ↑ Time production task | T3  T4  T5  T6  T7  T8 | 33.6  40.2  39,6  49.2  39.9  51.0 | 2.1  2.6  2.1  3.3  2.5  2.7 | 5 | 50.0  58.4  54.7  59.9  56.9  57.9 | 1.4  1.7  1.5  2.2  2.4  1.2 | 5 | 4.59 [2.23 - 6.96]*  4.14 [1.94 - 6.34]  4.14 [1.94 - 6.33]  1.91 [0.41 - 3.40]  3.47 [1.51 - 5.43]  1.65 [0.22 - 3.09] | (93) Table 2. Time production task (T3 to T8) |
| THC x Placebo | ↓ SCR | Stress  Post-Stress | 13.69  9.55 | 1.27  1.04 | 15 | 11.22  8.42 | 1.78  1.05 | 15 | 0.43 [0.30 - 1.15]  0.29 [0.43 - 1.01] | (94) Table 1. After intoxication. |
| Low THC x Placebo | ↑ Anxiety | 20 min  1 h 20 min  2 h 20 min | 15  14  14 | 4  4  4 | Not clearly described | 20  23  16 | 7  7  4 | Not clearly described | Not calculated | (95) Table 3. Anxious Rate. |
| High THC x Placebo | ↑ Anxiety | 20 min  1 h 20 min  2 h 20 min | 15  14  14 | 4  4  4 | Not clearly described | 40  30  29 | 6  6  8 | Not clearly described | Not calculated | (95) Table 3. Anxious Rate. |
| Low CBC and Low CBD x Placebo | ↑ Anxiety | 20 min  1 h 20 min  2 h 20 min | 15  14  14 | 4  4  4 | Not clearly described | 32  28  20 | 6  6  6 | Not clearly described | Not calculated | (95) Table 3. Anxious Rate. |
| Low CBC and High CBD x Placebo | ↑ Anxiety | 20 min  1 h 20 min  2 h 20 min | 15  14  14 | 4  4  4 | Not clearly described | 29  25  25 | 6  6  6 | Not clearly described | Not calculated | (95) Table 3. Anxious Rate. |
| High CBC and Low CBD x Placebo | ↑ Anxiety | 20 min  1 h 20 min  2 h 20 min | 15  14  14 | 4  4  4 | Not clearly described | 30  27  24 | 6  5  5 | Not clearly described | Not calculated | (95) Table 3. Anxious Rate. |
| High THC + Placebo x Low THC + Placebo | ↓ Anxiety | 00:20 | 0.00 | 2.03 | Not clearly described | -2.44 | 2.44 | Not clearly described | Not calculated | (95) Figure 1. Anxious Rating after THC pre smoking. |
| High THC + Placebo x Low CBC + Low CBD | ↑ Anxiety | 00:20 | 0.00 | 2.03 | Not clearly described | 24.90 | 10.60 | Not clearly described | Not calculated | (95) Figure 1. Anxious Rating after THC pre smoking. |
| High THC + Placebo x Low CBC + High CBD | ↑ Anxiety | 00:20 | 0.00 | 2.03 | Not clearly described | 6.95 | 11.83 | Not clearly described | Not calculated | (95) Figure 1. Anxious Rating after THC pre smoking. |
| Low THC + Placebo x Low CBC + Low CBD | ↑ Anxiety | 00:20 | -2.44 | 2.44 | Not clearly described | -0.82 | 10.21 | Not clearly described | Not calculated | (95) Figure 1. Anxious Rating after THC pre smoking. |
| Low THC + Placebo x Low CBC + High CBD | ↑ Anxiety | 00:20 | -2.44 | 2.44 | Not clearly described | 15.20 | 6.92 | Not clearly described | Not calculated | (95) Figure 1. Anxious Rating after THC pre smoking. |
| THC 15 mg x Placebo | ↑ Anxiety State | 3 h  4.5 h  5.5 h | 0 | Not showed | 9 | 4.63  4.21  6.29 | 1.58  1.99  3.57 | 9 | Not calculated | (96) Figure 2D. Anxiety State |
| THC 5 mg x Placebo | ↑ Anxiety State | 3 h  4.5 h  5.5 h | 0 | Not showed | 9 | 2.00  1.77  3.57 | 1.11  2.15  1.86 | 9 | Not calculated | (96) Figure 2D. Anxiety State |
| Sativex High (THC 16.2 mg + CBD 15 mg) x Placebo | ↑ Anxiety State | 3 h  4.5 h  5.5 h | 0 | Not showed | 9 | 3.59  6.61  5.81 | 1.84  3.16  3.05 | 9 | Not calculated | (96) Figure 2D. Anxiety State |
| Sativex Low (THC 5.4 mg THC + CBD 5 mg) x Placebo | ↔ Anxiety State | 3 h  4.5 h  5.5 h | 0 | Not showed | 9 | 1.05  1.30  2.02 | 1.88  3.17  1.22 | 9 | Not calculated | (96) Figure 2D. Anxiety State |
| THC 15 mg x Placebo | ↑ “Feeling anxious” on VAS | 2.5 h  3.5 h | 0 | Not showed | 9 | 6.26  6.29 | 3.57  2.73 | 9 | Not calculated | (96) Figure 2E. Anxious State |
| THC 5 mg x Placebo | ↑ “Feeling anxious” on VAS | 2.5 h  3.5 h | 0 | Not showed | 9 | 3.34  3.57 | 1.82  1.86 | 9 | Not calculated | (96) Figure 2E. Anxious State |
| Sativex High (THC 16.2 mg + CBD 15 mg) x Placebo | ↑ “Feeling anxious” on VAS | 2.5 h  3.5 h | 0 | Not showed | 9 | 6.05  5.81 | 3.43  3.05 | 9 | Not calculated | (96) Figure 2E. Anxious State |
| Sativex Low (THC 5.4 mg + CBD 5 mg) x Placebo | ↑ “Feeling anxious” on VAS | 2.5 h  3.5 h | 0 | Not showed | 9 | 6.05  5.81 | 3.43  3.05 | 9 | Not calculated | (96) Figure 2E. Anxious State |
| Sativex High (THC 16.2 mg + CBD 15 mg) x Sativex Low (THC 5.4 mg + CBD 5 mg) | ↓ “Feeling anxious” on VAS | 2.5 h  3.5 h | 6.05  5.81 | 3.43  3.05 | 9 | 6.05  5.81 | 3.43  3.05 | 9 | 0.54 [0.40 - 1.48]  0.58 [0.37 - 1.52]* | (96) Figure 2E. Anxious State |
| 2 mg (Low THC) x Placebo | ↓ Anxiety | -1 h  0 h  1 h  2 h  3 h  4 h  5 h  6 h | 0.67  0.85  1.02  0.70  0.66  0.57  0.67  0.59 | Not described | 4 | 1.19  1.05  0.83  0.69  0.66  0.57  0.63  0.65 | Not described | 4 | Not calculated | (97) Figure 4. POMS Anxiety. |
| 4 mg (Med THC) x Placebo | ↓ Anxiety | -1 h  0 h  1 h  2 h  3 h  4 h  5 h  6 h | 0.67  0.85  1.02  0.70  0.66  0.57  0.67  0.59 | Not described | 4 | 0.69  0.75  0.59  0.76  0.51  0.40  0.34  0.49 | Not described | 4 | Not calculated | (97) Figure 4. POMS Anxiety. |
| 5 mg (High THC) Placebo | ↑ Anxiety | -1 h  0 h  1 h  2 h  3 h  4 h  5 h  6 h | 0.67  0.85  1.02  0.70  0.66  0.57  0.67  0.59 | Not described | 4 | 0.69  0.78  0.83  0.80  0.77  0.77  0.84  0.81 | Not described | 4 | Not calculated | (97) Figure 4. POMS Anxiety. |
| Nabilone X Placebo | ↓ Anxiety | 4 d  11 d  18 d  32 d | 1.37  1.09  1.23  1,26 | Not described | 5 | 1.20  0.72  0.54  0.44 | Not described | 5 | Not calculated | (98) Figure 1. Hamilton Anxiety Rating Scale. |
| Nabilone X Placebo | ↓ Anxiety | 4 d  11 d  18 d  32 d | 1.73  1.33  1.55  1.64 | Not described | 5 | 1.65  0.67  0.47  0.40 | Not described | 5 | Not calculated | (98) Figure 2. SCL-56. |
| Clinical Assessment (CA) 1 x CA2 | ↓ CAPS arousal score | Pre THC treatment compared to Post THC treatment | 32.3 | 1.49 | 10 | 24.3 | 2.88 | 10 | 1.16 [0.21 - 2.11] | (99) Table 3. Average psychometric scores. |
| Clinical Assessment (CA) 1 x CA2 | ↓ NFQ frequency of nightmares | Pre THC treatment compared to Post THC treatment | 0.81 | 0.17 | 10 | 0.44 | 0.12 | 10 | 0.84 [0.08 -1.75] | (99) Table 3. Average psychometric scores. |
| Clinical Assessment (CA) 1 x CA2 | ↓ CGI-S | Pre THC treatment compared to Post THC treatment | 6.0 | 0.14 | 10 | 4.9 | 0.31 | 10 | 1.52 [0.52 - 2.52] | (99) Table 3. Average psychometric scores. |
| Clinical Assessment (CA) 1 x CA2 | ↓ CGI-I | Pre THC treatment compared to Post THC treatment | 3.5 | 0.16 | 10 | 2.7 | 0.33 | 10 | 1.01 [0.08 - 1.94] | (99) Table 3. Average psychometric scores. |
| Clinical Assessment (CA) 1 x CA2 | ↓ Pittsburgh Sleep Quality Index | Pre THC treatment compared to Post THC treatment | 17.20 | 0.83 | 10 | 13.9 | 1.41 | 10 | 0.95 [0.03 - 1.87] | (99) Table 3. Average psychometric scores. |
| Clinical Assessment (CA) 1 x CA2 | ↓ NES score | Pre THC treatment compared to Post THC treatment | 32.2 | 3.57 | 10 | 22.9 | 2.75 | 10 | 0.97 [0.05 - 1.75] | (99) Table 3. Average psychometric scores. |

Legend: ↑ = increase; ↓ = reduction; ↔ = no change; STAI = State Trait Anxiety Inventory; VAMS = Visual Analogue Mood Scale; VAS = Visual Analogue Scale; POMS = Profile of Mood State; SCR = skin conductance response; * indicasse the d value expressed in the text.
